# Supplementary material for: Males’ perceptions and practices towards maternity care in rural southeast Nigeria: Policy implication of participatory action research for safe motherhood
Source: PLoS One. 2023 Mar 30;18(3):e0282469. doi: 10.1371/journal.pone.0282469 (PMC10062587; doi:10.1371/journal.pone.0282469)
Supplement: Attachment — Submitted filename: Review.docx [file pone.0282469.s002.docx]

This manuscript describes an interventional study that utilizes multiple components of a community-participatory program to improve male partner involvement in maternal and newborn care. Given that it is established that male involvement is positively associated with improved reproductive health outcomes in Sub-Saharan Africa, high quality evidence-based interventions to inform practice and policy are needed. I submit the following comments:

Major comments:

Introduction and throughout the manuscript: Suggest replacing "husbands" with male partners. The use of "husbands/wives" connotes legal marriage, which may not necessary be the case for most individuals given the setting under consideration. Male partners may be more inclusive language.

Methods

Suggest including a flow chart depicting sampling and recruitment activities to make it easier for readers to understand.

Line 119 - 123: Suggest including numbers to describe the poor maternal health indices in Ebonyi state to give a general audience more contextual information. Please provide the source of these metrics.

Line 152: "Study instrument": It would be helpful if the authors included a copy of the survey instrument administered for data collection. If not feasible, please provide a summary of how the key variables were defined. For instance, how was awareness of safe motherhood assessed?

Given that the survey instrument was adapted from a previous study, did the authors assess the validity and reliability of the tool for this population? What was the Cronbach's alpha for this population?

Line 160 - 173: In describing the intervention, the authors reported information from a previous published study that was focused on improving birth preparedness and complication readiness through a behavioral change intervention. It would be helpful for readers to briefly explain and focus on the specific components of this intervention that are targeted at male partners to improve their perceptions and attitudes toward safe motherhood.

Was the goal to "train" the study participants or educate them? Please clarify.

Also, how many of the male partners received the intervention? How long were the educational sessions for? Please clarify. This would help readers determine reach and fidelity of the intervention.

There is no mention of the mean difference in the analytical section. How was it computed and for what purpose?

Results

Suggest summarizing the findings as narratives rather than using the variable names to report the results.

Discussion

In introducing the premise of the study, the authors alluded to the low involvement of men in maternal health, notably their absence during ANC visits. However, in this study, the authors found that a high proportion of men were engaged with the healthcare system from accompanying their female partners during ANC visits. This may have led to the high level of awareness of maternity care and safe motherhood among the respondents. It is also likely that the education the men received (if any) during the ANC visits may have improved some of the other domains. This should be discussed.

Line 271 - 273:...high mean scores at baseline for maternity care variables like...: In the discussion section, the focus should be on contextualizing these findings and not be repetitive of the results section.

Similar comments for Lines 287 and 304

Minor comments

I would suggest the authors review the manuscript critically for grammar and clarity.
